# Therapeutic mechanism of cord blood mononuclear cells via the IL-8-mediated angiogenic pathway in neonatal hypoxic-ischaemic brain injury

**DOI:** 10.1038/s41598-020-61441-0

**Published:** 2020-03-10

**Authors:** Kye Hee Cho, Jee In Choi, Jin-Ock Kim, Joo Eun Jung, Dong-Wook Kim, MinYoung Kim

**Affiliations:** 1 Department of Rehabilitation Medicine, CHA Gumi Medical Center, CHA University College of Medicine, Gumi, Gyeongsangbukdo Republic of Korea; 2 Rehabilitation and Regeneration Research Center, CHA University, Seongnam, Republic of Korea; 3 College of Pharmacy, Ajou University, Suwon, Gyeonggi-do Republic of Korea; 4 Department of Neurology, University of Texas Health Science Center at Houston, McGovern Medical School, Houston, Texas USA; 5 Department of Physiology, Yonsei University College of Medicine, Seoul, Republic of Korea; 6 Department of Rehabilitation Medicine, CHA Bundang Medical Center, CHA University College of Medicine, Seongnam, Gyeonggi-do Republic of Korea

**Keywords:** Medical research, Stem-cell research

## Abstract

In a clinical trial of cerebral palsy, the level of plasma interleukin-8 (IL-8) was increased, correlated with motor improvement, after human umbilical cord blood mononuclear cell (hUCBC) infusion. This study aimed to elucidate the role of IL-8 in the therapeutic effects of hUCBCs in a mouse model of hypoxic-ischaemic brain injury (HI). In P7 HI mouse brains, hUCBC administration at day 7 after HI upregulated the gene expression of Cxcl2, the mouse IL-8 homologue and increased the expression of its receptor, CXCR2. hUCBC administration restored the sequential downstream signalling axis of p-p38/p-MAPKAPK2, NFκB, and angiogenic factors, which were downregulated by HI. An *in vitro* assay revealed the downregulation of the angiogenic pathway by CXCR2 knockdown and p38 inhibition. *In vivo* p38 inhibition prior to hUCBC administration in HI mouse brains produced identical results. Behavioural outcomes revealed a therapeutic effect (*ps* < 0.01) of hUCBC or IL-8 administration, which was correlated with decreases in infarct size and angiogenic findings in the striatum. In conclusion, the response of the host to hUCBC administration in mice upregulated Cxcl2, which led to the activation of the IL-8-mediated p-p38 signalling pathway. The upregulation of the downstream pathway and angiogenic growth factors via NFκB can be inferred to be the potential therapeutic mechanism of hUCBCs.

## Introduction

Cerebral palsy is the most common cause of severe motor disability that exists throughout life from the early childhood period and is attributed to injury in the developing brain^1,2^. Perinatal hypoxic-ischaemic brain injury (HI) is the main pathology of cerebral palsy^3^. Although therapeutic hypothermia is a currently available treatment for near-term infants with HI^4^, the possibility of disability or death remains high, ranging from 31% to 55%^5^, requiring adjuvant therapy to improve the outcome. For the treatment of sequelae after HI, cell therapy with human umbilical cord blood mononuclear cells (hUCBC) has shown some efficacy in preclinical and clinical studies^6,7^. While several mechanisms underlying the effects of hUCBCs, including cytokine production^8^ have been suggested^9,10^, a recent clinical study revealed a significant increase in plasma interleukin-8 (IL-8) levels in hUCBC-treated subjects. The IL-8 elevation at 12 days after the treatment showed a definite correlation with the improvement of gross motor function 6 months later^11^.

Human IL-8 is a neutrophil chemoattractant that binds to transmembrane G-protein-coupled chemokine receptors 1 and 2 (CXCR1, 2)^12^. Multiple processes that are essential for angiogenesis, endothelial cell proliferation, and capillary tube organization are mediated by IL-8^13,14^. It is noteworthy that the response to injury in the developing brain is different from that in the adult brain with continuing inflammation, which exacerbates brain damage for years after the initial insult^15^. Thus, the implications of neuroprotection are more important for young HI victims, with a close relationship to angiogenesis^16^. IL-8 exerts a neuroprotective function by producing growth factors^17^, inhibiting apoptosis^18^, and modulating synaptic transmission^19^ in brain cells.

Based on previous studies on the stimulation of angiogenesis by stem cells via an IL-8-mediated pathway, a study was designed to determine the effect of hUCBCs on angiogenesis and neuroprotection in a mouse HI model. In the translation of the clinical study, mice at postnatal day 7 were selected to replicate a mean age of 3.5 years^11^ for hUCBC administration after HI. The following potential signalling molecules in the IL-8-related angiogenic pathway were included in the experiment: Cxcl2, a representative mouse homologue of IL-8; CXCR2, the transmembrane receptor for IL-8; p38 mitogen-activated protein kinase (MAPK); and the transcription factor nuclear factor kappa B (NFκB). It was found that VEGF-driven actin-based motility required p38 activation^20^ and that p38 inhibition enhanced abnormal endothelial hyperplasia in bFGF-induced neovascularization^21^. Additionally, the signalling of angiogenic genes such as VEGF, platelet-derived growth factor (PDGF), and bFGF was found to be related to NFκB^22,23^. In this study, IL-8-mediated p38 MAPK signalling and angiogenic gene expression following hUCBC administration were examined in HI mouse brain tissue *in vivo* and in mouse microvascular endothelial cells *in vitro*. The upregulation of the angiogenic genes and the endothelial marker CD31 in conjunction with NFκB activation was also investigated to establish the therapeutic mechanism of hUCBCs. In addition, the therapeutic effect of hUCBCs was evaluated through behavioural assessments and brain infarct size measurements in comparison with other treatments after HI. Thus, HI control, cyclosporine (CsA)-, hUCBC-, and IL-8-treated groups were included in this study. Cyclosporine was used as an adjuvant for the suppression of the host immune response in hUCBC-treated mice.

## Results

### Human UCB mononuclear cells upregulate CXCR2 and p38 phosphorylation and induce the subsequent degradation of IκB in the HI mouse brain

**Figure 1:**
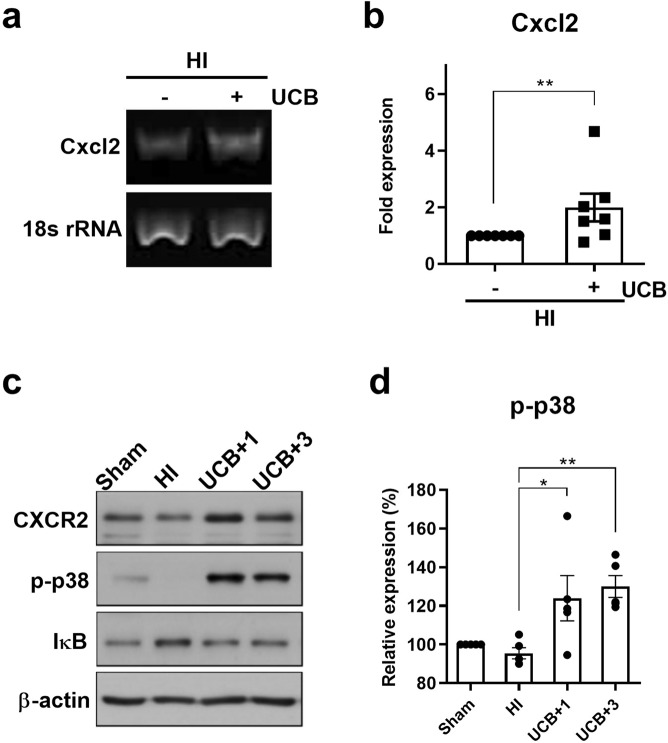
The effects of hUCBCs on the IL-8-mediated signalling pathway include the upregulation of Cxcl2 and CXCR2 expression, p38 activation, and subsequent degradation of IκB in the HI mouse brain. (**a**) The mRNA level of mouse Cxcl2, the IL-8 homologue was upregulated at 1 h post-hUCBC treatment according to RT-PCR analysis of the HI mouse brain (*n* = 5). (**b**) The upregulation of Cxcl2 expression after hUCBC administration in the HI mouse brain was consistent and significant according to real-time PCR assays 1 h post-hUCBC injection (*n* = 7). (**c**) The levels of mouse CXCR2 (the receptor for Cxcl2), p-p38, IκB, and ß-actin in the sham, HI, and hUCBC groups sacrificed on post-hUCBC days 1 and 3 were assayed using Western blotting to observe the consecutive changes after Cxcl2 upregulation. The level of CXCR2 was upregulated in parallel with p38 activation and the successive degradation of IκB after hUCBC administration. (**d**) The graph depicts the band intensity in p-p38 Western blotting. The data were obtained from triplicate results. Data are shown as the mean ± SEM (*n* = 5). Asterisks (*) indicate significant differences (*p < 0.05, **p < 0.01, one-way ANOVA) between groups. HI, hypoxic-ischaemic brain injury; hUCBCs, human umbilical cord blood mononuclear cells; UCB, HI mice subjected to hUCBC treatment.

In mice, the gene encoding IL-8 has been deleted^24^, and murine Cxcl2 is a functional homologue of IL-8. Mice exhibit a receptor homologous to human CXCR2 that is able to mediate signal transduction in response to human IL-8^25,26^. To determine whether murine Cxcl2 and its receptor, CXCR2, are upregulated by hUCBC administration in a mouse HI model, RT-PCR and Western blotting were performed. The Cxcl2 mRNA level was increased in the brains of HI mice subjected to hUCBC injection according to RT-PCR results (Fig. 1a). The upregulation of Cxcl2 expression 1 h after hUCBC injection was consistently noted via real-time PCR (*p* < 0.01) (Fig. 1b), and it continued until 1 day after injection (data not shown). In addition, the expression levels of CXCR2 and its downstream kinase p-p38 were upregulated following the upregulation of Cxcl2 by hUCBC administration in the HI model (Fig. 1c,d).

Most MAPK and phosphoinositide 3-kinase (PI3K) pathways are reported to be neuroprotective signalling pathways^27^. Within these pathways, extracellular signal-regulated kinase (Erk)-MAPK 1/2, the PI3K effector serine/threonine kinase Akt, and p38 MAPK are suggested to transduce IL-8 signals in angiogenesis^28–30^. Therefore, p-Erk 1/2 (T202/Y204), p-Akt (S473), and p-p38 (T180/Y182) were monitored as potential components of IL-8-mediated signalling pathways. The results showed that p-p38 (T180/Y182), which was initially downregulated after HI, was increased significantly in response to hUCBC treatment (*p* < 0.05) (Fig. 1c,d); however, p-Erk 1/2(T202/Y204) and p-Akt (S473) did not show any substantial change (Supplementary Fig. S1).

NFκB is involved in the downstream pathway of p38-mediated angiogenesis^31^. To evaluate whether NFκB is activated in response to p38 phosphorylation, the level of IκB was examined by Western blotting. The level of p-p38 was decreased after HI but was significantly increased by hUCBC administration (*p* < 0.05*)*, whereas IκB showed the greatest increase during HI and then decreased after hUCBC administration in mouse hypoxic brain tissues (Fig. 1c,d). These data indicate the proximity of the p-p38 and NFκB pathways in the response to hUCBC administration and support the notion that the activation of the p38 pathway leads to subsequent NFκB activation via IκB degradation as a result of hUCBC administration.

### Human UCB mononuclear cells upregulate the synthesis of the angiogenic growth factors VEGF, bFGF, and PDGF and the endothelial marker CD31 in the HI mouse brain

**Figure 2:**
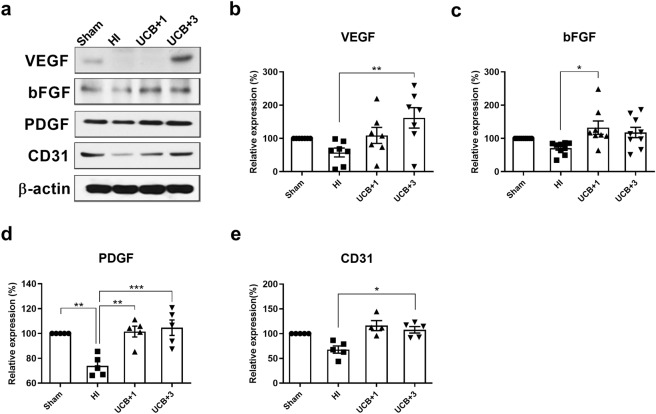
Effects of hUCBCs on the IL-8-mediated upregulation of angiogenic growth factors and the endothelial marker CD31. The affected brain hemispheres of HI mice sacrificed 1 day after hUCBC administration were collected for Western blot analysis. (**a**) The protein levels of angiogenic growth factors VEGF, bFGF, and PDGF, and the endothelial marker CD31 were all downregulated after HI and then significantly recovered after hUCBC administration according to Western blotting. (**b**–**e**) The graphs depict the band intensity in each Western blot assay. Data are shown as the mean ± SEM (*each n* ≥ 5). The data were collected from triplicate results. Asterisks (*) indicate significant differences (*p < 0.05, **p < 0.01, ***p < 0.001, one-way ANOVA). HI, hypoxic-ischaemic brain injury; hUCBC, human umbilical cord blood mononuclear cells; UCB, HI mice subjected to hUCBC treatment.

To confirm whether hUCBC administration upregulates angiogenesis in the brains of HI mice, the protein levels of VEGF, bFGF, PDGF, and CD31 were measured by Western blotting. All angiogenic growth factors that were initially downregulated after HI, were upregulated beginning on the first day after hUCBC injection (Fig. 2a–d). Significantly increased protein levels of PDGF were maintained up to 3 days post-hUCBC (*p* < 0.01), and the levels of VEGF and the endothelial marker CD31 increased significantly at 3 days post-hUCBC administration (*ps* < 0.05) (Fig. 2).

### CXCR2 knockdown using siRNA transfection downregulates the IL-8-mediated angiogenic pathway *in vitro*

**Figure 3:**
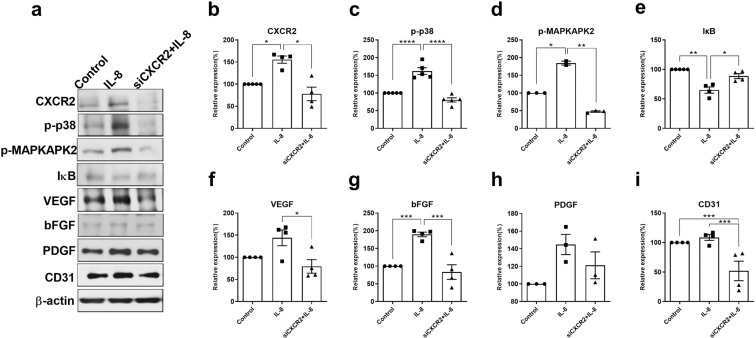
CXCR2 knockdown via siRNA transfection in brain endothelial bEnd.3 cells resulted in reduced activation of the downstream angiogenic pathway. Brain endothelial bEnd.3 cells cultured in DMEM for 24 h were transfected with siRNA targeting CXCR2 (90 pmol) and incubated for 24 h. (**a**) CXCR2 knockdown resulted in the downregulation of IL-8-downstream p-p38, angiogenic growth factors, IκB degradation representing NFκB release, and CD31 (endothelial marker) according to Western blotting, despite IL-8 treatment. (**b–i**) The graphs depict the band intensity in each Western blot assay. Data are shown as the mean ± SEM (*each n* ≥ 3). The data were obtained from triplicate results. Asterisks (*) indicate significant differences (*p < 0.05, **p < 0.01, ***p < 0.001, ****p < 0.0001, one-way ANOVA). IL-8, bEnd.3 cells treated with IL-8 without prior CXCR2 knockdown; siCXCR2+IL-8, CXCR2-knockdown bEnd.3 cells treated with IL-8.

To confirm the involvement of the IL-8 receptor CXCR2 in the angiogenic cascade of downstream of IL-8, mouse brain endothelial bEnd.3 cells were transfected by CXCR2 siRNA. In the CXCR2-knockdown bEnd.3 cells, the IL-8-downstream molecules p-p38, p-MAPKAPK2, the angiogenic growth factors VEGF and bFGF, and the endothelial marker CD31 were all downregulated according to Western blot analysis, despite IL-8 treatment (*p* < 0.05) (Fig. 3a–i). The degradation of IκB, reflecting NFκB activation, was also measured to observe the effect on IL-8 in CXCR2-knockdown bEnd.3 cells. The activation of NFκB was found to be prevented by CXCR2 knockdown. Taken together, these findings may indicate that CXCR2 is responsible for IL-8 mediated angiogenesis through NFκB regulation.

### Inhibition of p38 prior to hUCBC administration prevents angiogenic growth factors upregulations and angiogenesis by hindering the downstream p38 pathway in the HI mouse brain

To evaluate whether the increased activity of p38 induced by hUCBC administration is involved in the upregulation of angiogenic growth factors, SB203580 was employed for p38 inhibition *in vivo*. SB203580 is a specific inhibitor of p38α and p38β that suppresses the p38 MAPK stimulation of MAPKAPK2.

**Figure 4:**
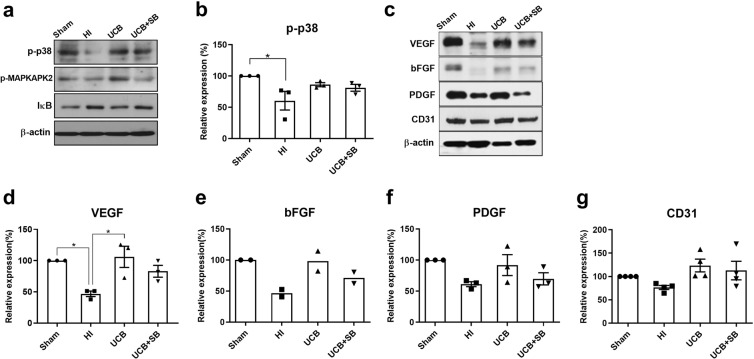
*In vivo* effects of p38 inhibition on the IL-8-mediated angiogenic pathway in the HI mouse brain. (**a**) Mice treated with the p38 inhibitor SB203580 (0.25 mg/kg) 30 min prior to hUCBC injection showed a decrease in the levels of p-MAPKAPK2 and IκB in response to hUCBC treatment, despite an increase in p-p38 after hUCBC injection according to Western blotting. (**b**) The graph depicts the band intensity of p-p38. (**c**) The protein levels of the angiogenic growth factors VEGF, bFGF, and PDGF and the endothelial marker CD31 were all downregulated following p38 inhibition according to Western blotting. (**d–g**) The graphs depict the band intensity in each Western blot assay. Data are shown as the mean ± SEM (*each n* ≥ 3). The data shown in the graphs were obtained from triplicate results. Asterisks (*) indicate significant differences (*p < 0.05, one-way ANOVA). HI, hypoxic-ischaemic brain injury; hUCBCs, human umbilical cord blood mononuclear cells; UCB, HI mice subjected to hUCBC treatment; UCB+SB, mice treated with SB203580 prior to hUCBC treatment.

The phosphorylation of MAPKAPK2 induced by hUCBCs was blocked in mouse brain tissue using SB203580 as a p38 inhibitor. The level of IκB, which is degraded to release NFκB for intranuclear transfer, showed a tendency to increase after p38 inhibition, suggesting that NFκB activation is regulated by the p38 pathway (Fig. 4a,b). Western blot results for the angiogenic growth factors VEGF, bFGF, and PDGF and the direct endothelial marker CD31 showed a tendency to be downregulated in the brain tissue of mice treated with the p38 inhibitor SB203580 prior to hUCBC administration (Fig. 4c–g). These *in vivo* p38 inhibition data suggest that hUCBC administration induces angiogenesis mediated by a p38-dependent pathway.

### Inhibition of p38 in mouse endothelial cells prevents the IL-8-mediated upregulation of angiogenic factors *in vitro*

**Figure 5:**
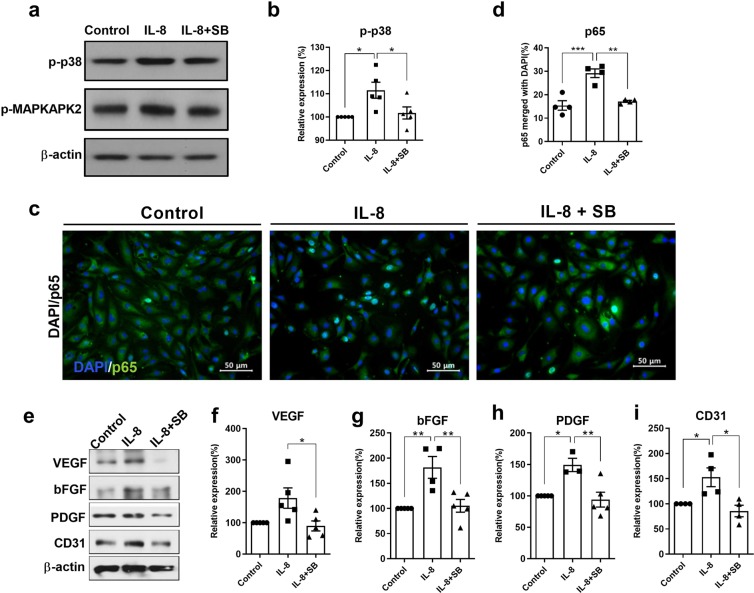
Effects of p38 inhibition *in vitro* on IL-8-mediated angiogenic growth factor expression in mouse brain vascular bEnd.3 cells. (**a**) A significant increase in p-p38 was noted after IL-8 treatment (50 ng/ml), while SB203580 (50 μM) application 30 min prior to IL-8 administration decreased p-MAPKAPK2 levels according to Western blot analysis of OGD-conditioned bEnd.3 cells. (**b**) The graph depicts the band intensity of p-p38 in the Western blot assay. Data are shown as the mean ± SEM (*each n* = 5). (**c**) The proportion of merged p65- and DAPI-stained cells was significantly greater at 6 h after IL-8 treatment than in the OGD-only control cells and p38-inhibited cells upon immunocytochemistry analysis (200×). (**d**) The graph depicts the proportion of merged p65 and DAPI-stained cells. Data are shown as the mean ± SEM (*each n* = 4). (**e–i**) Westerns blot analysis of cells harvested 12 h after IL-8 treatment showed the upregulation of VEGF, bFGF and PDGF and the endothelial marker CD31, whereas cells pretreated with SB203580 exhibited the downregulation of the angiogenic growth factors and CD31, as shown in the graphs depicting the band intensity. Data are shown as the mean ± SEM (*each n* ≥ 4). The data shown in the graphs were collected from triplicate results. Asterisks (*) indicate significant differences (*p < 0.05, **p < 0.01, one-way ANOVA) according to Tukey’s multiple comparisons between groups. OGD, oxygen-glucose deprivation; Green, p65; DAPI, 4′, 6-diamidino-2-phenylindole for nuclear staining; Control, bEnd.3 cells without IL-8 treatment; IL-8+SB, SB203580 infused prior to IL-8 administration.

To confirm the above *in vivo* results, the p38 inhibitor SB203580 was applied to mouse brain endothelial bEnd.3 cells. In response to human IL-8 treatment, p38 was immediately phosphorylated with a strong peak of phosphorylation being observed at 3 hours, and the phosphorylation signal was sustained up to 6 h after stimulation (data not shown). As shown in Fig. 5, the phosphorylation of p38 was significantly increased in bEnd.3 cells harvested 3 h after IL-8 treatment (*p* < 0.05), while SB203580 reduced the phosphorylation of p38 and MAPKAPK2. In addition, the nuclear translocation of NFκB was increased and maintained for 6 h in oxygen-glucose deprivation (OGD)-conditioned cells following IL-8 treatment (Supplementary Fig. S2) (*p* < 0.05), whereas this response was inhibited by SB203580 according to immunocytochemistry (*p* < 0.05) (Fig. 5c,d). A significant reduction in angiogenic growth factors and CD31 was observed after p38 inhibition 30 min prior to IL-8 administration according to Western blotting (*ps* < 0.05) (Fig. 5e–i). Taken together, the data indicate that the IL-8-mediated p38 pathway induces angiogenesis *in vivo*, which was confirmed *in vitro* using vascular endothelial cells.

### Upregulation of angiogenesis is observed in mouse brains treated with hUCBCs or IL-8 upon immunohistochemistry *in vivo*

**Figure 6:**
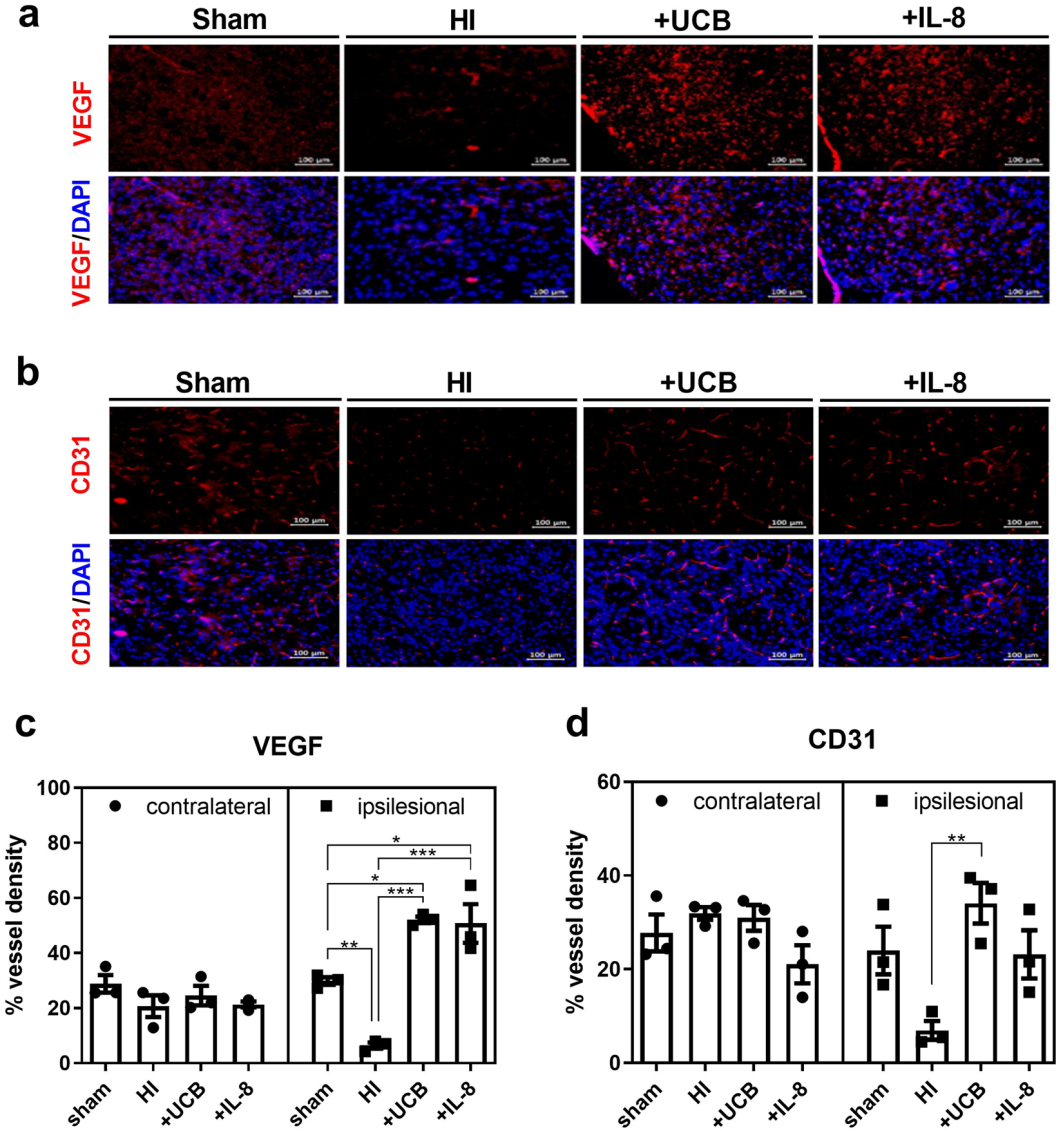
Effects of hUCBCs and IL-8 on angiogenesis *in vivo* in the HI mouse brain. Angiogenesis was observed *in vivo* through immunohistochemistry staining for VEGF and CD31 in the peri-infarct cortex and striatum of HI mice dissected one week after either hUCBC (3 × 10^7^/kg) or IL-8 (50 µg/kg) injection. (**a**,**c**) The graph depicts the density of vessels (percentage) expressing VEGF among all cells stained with DAPI according to IHC analysis in the ipsilesional and contralateral hemispheres. (**b**,**d**) The graph depicts the percentage of vessel density expressing CD31 among DAPI-stained cells observed in the lesion side and unaffected contralateral hemispheres. On the other hand, the unaffected contralateral brain hemisphere was not significantly affected by HI, hUCBC or IL-8 administration. IHC findings of the contralateral hemisphere are depicted in Supplementary Fig. S3. Data are shown as the mean ± SEM (*each n* = 3). Asterisks (*) indicate significant differences (*p < 0.05, **p < 0.01, ***p < 0.001, one-way ANOVA) according to Tukey’s multiple comparisons between groups. HI, hypoxic-ischaemic brain injury; DAPI, 4′, 6-diamidino-2-phenylindole for nuclear staining; +UCB, HI mice subjected to hUCBC treatment; +IL-8, HI mice subjected to IL-8 treatment; hUCBCs, human umbilical cord blood mononuclear cells.

The density of vessels (percentage) expressing VEGF and CD31 was markedly decreased in the HI mouse brain, which was recovered one week after either hUCBC or IL-8 treatment. The density of vessels expressing VEGF was increased significantly by both hUCBC and IL-8 injection (*p* < 0.001) compared to the HI control, whereas CD31 expression was increased significantly only by hUCBC administration (*p* < 0.01) (Fig. 6). The angiogenic effect of hUCBCs was confined to the affected brain hemisphere. The density of vessels in the unaffected contralateral hemisphere of mice in the sham group was not significantly different compared with that in the other groups (Supplementary Fig. S3).

These results were supported by *in vitro* experiments in bEnd.3 cells showing an increase in the MMP9 enzymatic activity signal (greatest under 50 ng/ml IL-8 treatment) and tube formation after IL-8 treatment (Supplementary Figs. S4 and S5).

### Functional improvement in hUCBC or IL-8-treated HI mice occurred in parallel with improved cell viability and a reduced of infarct volume

**Figure 7:**
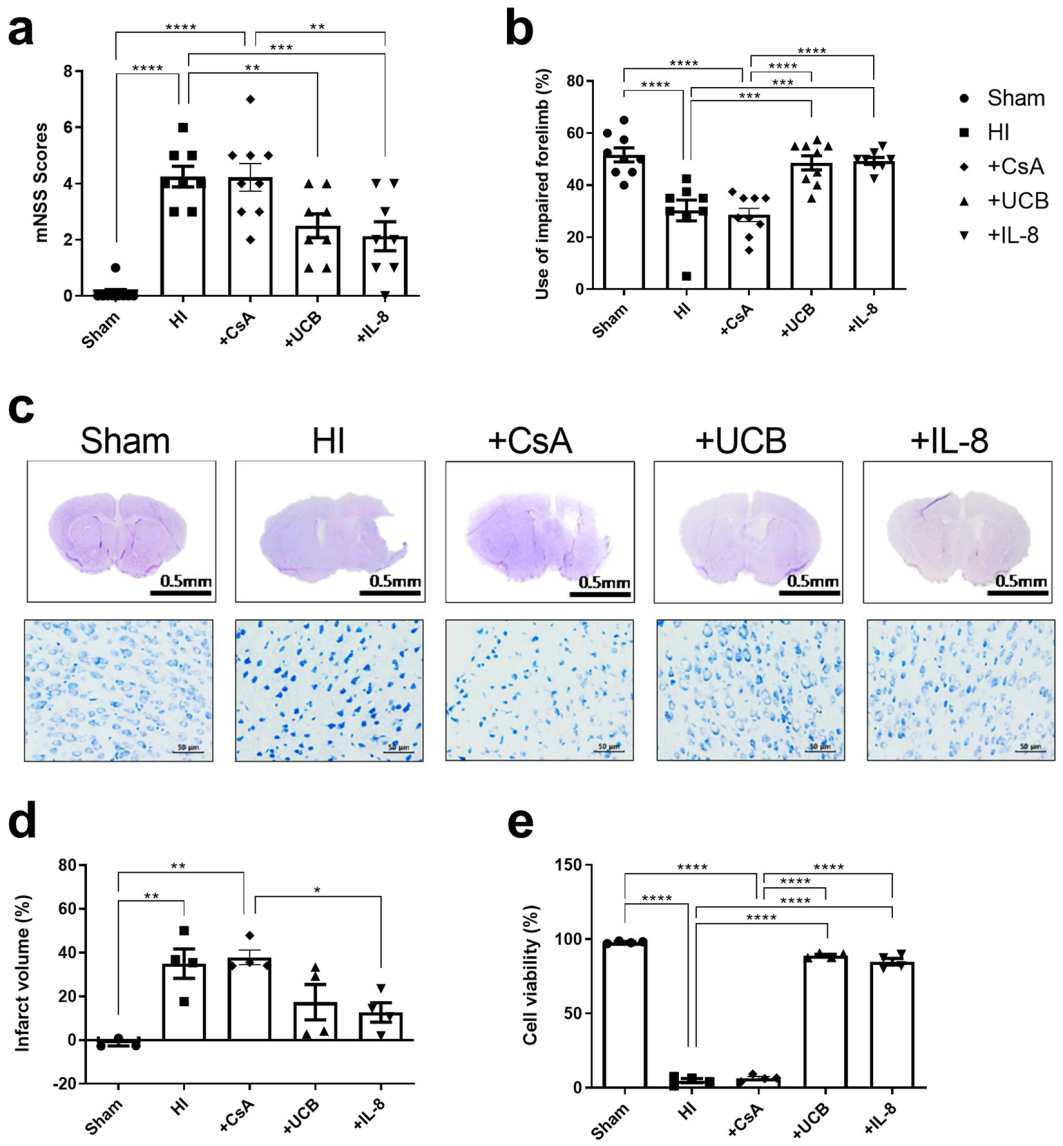
Behavioural improvement after hUCBC or IL-8 injection parallels the attenuation of brain damage. HI mice treated with hUCBCs or IL-8 showed significant improvement of the mNSS and cylinder tests at P42 in multiple comparisons with the sham, HI, and CsA-only groups. (**a**,**b**) The graphs depict mNSS scores and the percentage of impaired forelimb use in each group. Multiple comparisons of each group revealed no difference between the HI and CsA groups or among the sham, hUCBC and IL-8 groups. Data are shown as the mean ± SEM (*each n* ≥ 8). (**c**) Cresyl violet-stained brain coronal sections (above) were used for cell viability assessment of the affected brain cortex (below). (**d–e**) Each graph depicts the immunohistochemistry analysis of the infarct volume and viable cell counts. The reduction in infarct volume in the affected hemisphere was estimated in comparison to that in the contralateral side. Data are shown as the mean ± SEM (*each n* ≥ 3). Asterisks (*) indicate significant differences (*p < 0.05, **p < 0.01, ***p < 0.001, ****p < 0.0001, one-way ANOVA) according to Tukey’s multiple comparisons between groups. mNSS, modified neurological severity score; HI, hypoxic-ischaemic brain injury; hUCBCs, human umbilical cord blood mononuclear cells; +CsA, HI mice subjected to cyclosporine-only treatment; +UCB, HI mice subjected to hUCBC treatment; +IL-8, HI mice subjected to IL-8 treatment.

Functional performance assessed by using the modified neurological severity score (mNSS) and the cylinder test showed significant improvement in the hUCBC- or IL-8-treated groups compared with the HI-only control and CsA groups at 7 weeks after HI (*ps* < 0.01) (Fig. 7a,b). These results were consistent with the attenuation of brain damage *in vivo*. Cresyl violet staining of the brains of mice sacrificed after behavioural tests revealed a substantial decrease in the infarct volume as well as significantly greater cell viability in the hUCBC- and IL-8-treated groups comparison with the HI and CsA groups (*ps* < 0.0001) (Fig. 7c–e).

## Discussion

In this study, we demonstrated that hUCBCs induced the activation of an IL-8-mediated angiogenic cascade in a mouse HI model. Following hUCBC administration, the upregulation of the IL-8 homologue Cxcl2 and its receptor CXCR2, the phosphorylation of the downstream molecules p38 and MAPKAPK2, the degradation of IκB, leading to NFκB activation, and the upregulation of angiogenic growth factors and the endothelial marker CD31 all occurred in a consecutive manner beginning on the next day. The therapeutic effect of hUCBCs was ascertained according to behavioural outcomes 35 d later, and the results paralleled the *in vivo* attenuation of brain injury.

Human IL-8 is a small, soluble peptide (8–10 kDa) secreted by nonimmune cells as well as monocytes and macrophages^32^. In mice, two functional homologues of human IL-8 (keratinocyte-derived chemokine (Cxcl1) and macrophage inflammatory protein (Cxcl2)) are present^24–26,33,34^. Cxcl2 and CXCR2 were evaluated as the mouse IL-8 homologue and the receptor for the IL-8 family, respectively, in this study. hUCBC administration resulted in an increase in the Cxcl2 levels in HI mice, similar to the increase in the IL-8 levels observed in hUCBC-treated patients, which was correlated with motor function improvement^11^. Murine Cxcl2 elevation in the HI mouse brain after intraperitoneal hUCBC administration suggests a host response via the secretion of Cxcl2 by mouse cells. The host response of Cxcl2 production and downstream angiogenic cascade activation in the brain could be the driving forces in hUCBC therapy.

A systemic host response to the infusion of hUCBCs has also been reported in other studies. In Alzheimer’s disease model mice, the intravenous infusion of hUCBCs increases the levels of anti-inflammatory cytokines in plasma and reduces the level of circulating sCD40L, an important mediator of inflammation^35^. In a rat stroke model study, intravenous hUCBC injection induced a strong immunomodulatory reaction in the host via inhibiting splenic cytotoxic T cell release in association with an increase in anti-inflammatory IL-10 secretion and a reduction in pro-inflammatory cytokine levels^36^.

The receptor for IL-8 or Cxcl2, CXCR2 is present on the surface of endothelial cells^37^ and other types of cells, including leukocytes^38^, neurons^39^, and oligodendrocytes^40^. Human intestinal epithelial cells expressing CXCR2 show an IL-8-mediated angiogenic response^41^. In this study, the upregulation of CXCR2 was consistently observed following hUCBC administration *in vivo* or IL-8 treatment *in vitro* (Supplementary Fig. S6). Moreover, CXCR2-knockdown bEnd.3 cells exhibited the downregulation of all downstream components of the IL-8-mediated angiogenic pathway despite IL-8 treatment. This finding suggests that CXCR2 is responsible for the angiogenic effect of Cxcl2 (Fig. 3).

Among the pathways downstream of IL-8, the p38 MAPK pathway showed greater activation than the Erk or Akt pathway (Supplementary Fig. S1). The p38 MAPK pathway has been found to play an important role in endothelial cell migration^42^, which is essential for angiogenesis, and the inhibition of p38 attenuated VEGF-induced cell migration^20^. Similarly, the present study revealed that hUCBC administration upregulated the expression of the p38-downstream molecule p-MAPKAPK2 and angiogenic growth factors, while p38 inhibition downregulated these responses to a level comparable to that observed under HI (Fig. 4). In addition to its angiogenic effects, p38 activation has been reported to prompt neuronal differentiation^43,44^, axonal growth, dendritic differentiation, and increased neuronal survival^45^.

As a downstream target of the p38 pathway^31^, NFκB activation was followed in the present study. NFκB regulates the degradation of the vascular basement membrane and the remodelling of the extracellular matrix, which is essential for angiogenesis^46,47^. The administration of hUCBCs reduced the level of IκB, indicating that NFκB activation was promoted by hUCBCs (Fig. 1c). Prior p38 inhibition increased the IκB level, implying that the activation of NFκB is dependent on the p38 pathway (Fig. 4a).

The changes in the protein levels of the angiogenic growth factors VEGF, PDGF, and bFGF in response to HI, hUCBC or IL-8 treatment were consistent in brain tissue and cell culture experiments (Figs. 2–6). The decline in angiogenic growth factor expression induced by p38 inhibition was more prominent in endothelial cells *in vitro* than in brain tissue *in vivo*. This difference could be caused by differences in the environment, such as variable cellular components of brain tissues, rather than the bEnd.3 cell culture conditions alone. Alternatively, the vascular nature of bEnd.3 cells may have led to a stronger response.

One of the concerns in reproducing the corresponding clinical intervention in an experimental setting is related to the coadministration of immunosuppressant CsA^48^. To rule out any effect caused by CsA on therapeutic efficacy of hUCBCs or IL-8, we added another control group of HI mice treated only with CsA, which presented striking similarity to the HI group in contrast to the hUCBC or IL-8 groups in a multiple comparison analysis (*ps* < 0.01) (Fig. 7). This finding suggests that CsA has little influence in the course of hUCBC-prompted angiogenesis or on therapeutic efficacy in clinical practice.

Finally, the ultimate therapeutic effect of hUCBCs or IL-8 was revealed by behavioural improvements with ameliorated brain damage, accompanied by increased angiogenic findings in the HI mouse brain. Furthermore, cell viability and blood vessel densities were increased at 35 d post-therapy, accompanied by functional improvements under both hUCBC and IL-8 treatments. The levels of angiogenic growth factors and CD31 were increased by hUCBC treatment to the same or greater extent compared with IL-8 treatment in the mouse brain.

The current study has some limitations that need to be addressed in future research. First, the specific mechanism or cellular sources responsible for Cxcl2 elevation need to be determined. Second, it needs to be clarified which cells in the brain respond to IL-8 in humans or to its homologue in mice. Since the brain is a highly complicated organ consisting of various cell types, the specific cells that exert neuroprotective effects among these cells should be further investigated. It has been reported that oligodendrocyte progenitor cells are protected from apoptosis via CXCR2 signalling^49^. Similarly, CXCR2 inhibits β-amyloid-induced hippocampal neuronal apoptosis^50^. Third, the relationship between Cxcl2 elevation and CXCR2 upregulation and the subsequent increase in p-p38 following hUCBC administration needs to be clarified to delineate the molecular pathway upstream of p38. A study involving CXCR2 gene knockout mice would be helpful to provide further evidence in this regard.

In conclusion, this study confirmed that the administration of hUCBCs after HI upregulates angiogenic gene expression and resultant angiogenesis via the Cxcl2-mediated activation of the p38 pathway as a host response. Taken together with the previous clinical study, these results indicate that hUCBC-induced IL-8-mediated angiogenesis could be a key neuroprotective mechanism in the human HI brain.

## Materials and Methods

Human umbilical cord blood was supplied by the CHA Medical Center cord blood bank. The cryopreservation of donated hUCBCs was performed following the protocol of the facility; white blood cell concentrates were generated via the Rubinstein method and treated with dimethylsulfoxide and dextran before freezing in a controlled-rate freezer, then cryopreserved in a freezer at −198° within 36 hours after collection^51^. All human-related protocols were approved by the Institutional Ethics Committee of CHA Bundang Medical Center. The number of total nucleated cells injected was 3 × 10^7^/kg based on clinical trials in which this number of cells was used^7^. Briefly, the cells were prepared according to our protocol, in which the hUCBCs were thawed and then washed twice with a 1:1 solution of 5% albumin and 10% dextran to eliminate dimethyl sulfoxide^51^. The cells were subsequently dissolved in saline for injection.

### Animals

To assess the effect of hUCBCs on the IL-8-mediated angiogenic pathway, newborn Institute of Cancer Research (ICR) mice of both sexes were used. All experimental protocols were approved by the Institutional Animal Care and Use Committee of CHA University (Approval no. 160079, 180181). All mice were housed and maintained according to the regulations of the committee.

The HI model was adapted from the Rice-Vannucci model^52^ with minor modifications. Briefly, at postnatal day 7 (P7), the right carotid artery of the HI mice was ligated with 5–0 nylon blue under isoflurane anaesthesia. Then, the mice were placed in a warm chamber, which was perfused with a hypoxic gas mixture (8% oxygen, 92% nitrogen) for 1 h. The mortality rate of the mice was approximately one in 30 mice. The extent of the lesion was assessed through the semitransparent skull following a scalp incision under isoflurane anaesthesia on P13. Mice exhibiting severe injury involving more than half of the ipsilateral hemisphere and those without any injury were excluded^53^. Therefore, mice with mild to moderate injury were included in the analysis.

The study groups included the sham, HI, hUCBC treatment, and IL-8 treatment groups. In the hUCBC group, HI mice were treated with hUCBCs (3 × 10^7^ total nucleated cells/kg) at P14, 7 days post-HI. The immunosuppressant CsA (10 mg/kg) was administered to the hUCBC group for 5 consecutive days beginning on P13, in place of the clinical procedures, and the same protocol was followed for the other experiments using hUCBCs. A single injection of 50 µg/kg IL-8 was given once to HI mice at P14 on the same day as the hUCBC group. All materials injected into the mice were administered via the intraperitoneal route.

For evaluation of the sequential short-term molecular responses after hUCBC injection, ICR mice were randomly allocated to sham (*n* = 5), HI (*n* = 5), and hUCBC treatment (*n* = 10) groups. The HI group and sham group were sacrificed at P13, and the hUCBC group mice were sacrificed at P15 (*n* = 5) or P17 (*n* = 5) to observe the changes after hUCBC administration (Figs. 1 and 2).

For the *in vivo* p38 inhibition experiment, additional HI mice were randomly assigned to the control (*n* = 4) and hUCBC (*n* = 8) groups. Half of the hUCBC group received the p38 inhibitor SB203580 (Cell Signaling Technology, MA, USA; 0.25 mg/kg) 30 min prior to hUCBC administration. Dimethyl sulfoxide (0.01%) was used to dilute SB203580, and the same amount of dimethyl sulfoxide was injected into the hUCBC group without p38 inhibition. All groups for p38 inhibition study were sacrificed at P17 (Fig. 4).

Other HI mice were randomly assigned to the control (*n* = 4), IL-8 (*n* = 4) or hUCBC (*n* = 4) treatment groups for visualization of *in vivo* angiogenesis through IHC staining. One week after this treatment, their brains were dissected for IHC (Fig. 6).

**Figure 8:**
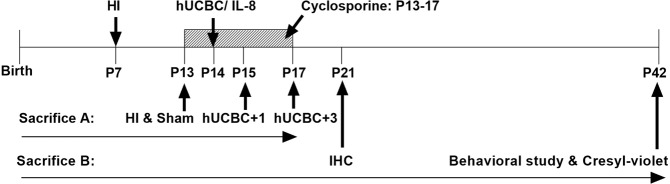
Schematic illustration of the timeline for the assessment of the effects of hUCBCs in ameliorating mouse brain lesions and behavioural outcomes. Postnatal day 7 (P7) ICR mice were conditioned for hypoxic-ischaemic brain injury. Intraperitoneal cyclosporine was administered to the hUCBC group and cyclosporine-only group beginning on P13 for 5 consecutive days (small arrows). At P14, the hUCBC group received hUCBCs (3 × 10^7^/kg) intraperitoneally, whereas IL-8 group received IL-8 (50 µg/kg) intraperitoneally. For long-term follow-up, mice were allocated to the sham, HI, cyclosporine, hUCBC, and IL-8 groups. At P21, the sham, HI, hUCBC, and IL-8 groups were assessed for angiogenesis via IHC. At P42, behavioural test of the mice was performed prior to sacrifice. Sacrifice A: sacrifice schedule for inhibition tests; Sacrifice B: for IHC and behavioural studies; HI, hypoxic-ischaemic brain injury; hUCBC, human umbilical cord blood mononuclear cells; IHC, immunohistochemistry.

For behavioural assessment, another set of newborn mice was randomly assigned to the sham (*n* = 9), HI control (*n* = 8), CsA (*n* = 9), hUCBC (*n* = 9), and IL-8 (*n* = 8) treatment groups. Immediately after the behavioural assessments conducted at P42 (35 d after HI), all groups were sacrificed to evaluate the infarct volume and cell viability in the affected brain hemisphere (Fig. 7). A schematic timeline of the HI treatments, including hUCBC injection, is presented in Fig. 8.

### Reverse transcription polymerase chain reaction (RT-PCR) and real-time PCR

The injured right hemispheres of the mouse brains were harvested and homogenized with TRIzol (Invitrogen) to extract total cellular RNA. ReverTra Ace qPCR RT Master Mix (Toyobo Co., Osaka, Japan) was used for cDNA synthesis. The primers for Cxcl2, a mouse homologue of IL-8, were Cxcl2 forward, 5′-CTCTCAAGGGCGGTCAAAAAGTT-3′, and Cxcl2 reverse, 5′-TCAGACAGCGAGGCACATCAGGTA-3′. Quantitative real-time PCR was performed in a CFX Connect Real-Time PCR Detection System (Bio-Rad Laboratories, Hercules, CA, USA). The amount of RNA used was 100 ng.

### Western blotting

Proteins were extracted from the affected hemisphere of the mouse brain using RIPA Lysis and Extraction Buffer (Thermo Fisher). The protein content of the brain tissue was quantified via the Bradford method according to the manufacturer’s instructions (Bio-Rad). Equal amounts (40 µg) of the samples were dissolved in SDS sample buffer and heated at 99 °C for 6 min. The samples were separated using SDS-PAGE and transferred to polyvinylidene difluoride membranes (Millipore, Bellerica, CA, USA). A 5% skimmed milk solution was used to block the membranes for 1 h at room temperature on a rocker. The housekeeping gene β-actin was employed as a loading control. Primary antibodies against IκB, bFGF, PDGF, and β-actin were purchased from Santa Cruz Biotechnologies (Santa Cruz, CA, USA). Antibodies against phosphorylated (p)-p38, p-MAPKAPK2, and p65 (NFKB) were purchased from Cell Signalling Technology (Danvers, MA, USA), while antibodies against CXCR2 and CD31 were purchased from Abcam (Cambridge, UK). The antibody for VEGF was purchased from Invitrogen and Novus Biologicals (Centennial, CO, USA). The primary target antibodies were all diluted 1:1,000 and incubated with the membranes at 4 °C for 16 h (overnight). A horseradish peroxidase-conjugated anti-rabbit IgG antibody (Santa Cruz) at a dilution of 1:20,000 or an anti-mouse IgG antibody (KPL, Inc., Gaithersburg, MD, USA) at a dilution of 1:10,000 was added to the corresponding primary antibodies, followed by incubation for 1 h at room temperature. The membranes were developed with the ECL reagent (Millipore).

### Cells and cultures

*In vitro* cell cultures were generated using immortalized mouse brain endothelial bEnd.3 cells, purchased from the ATCC (Manassas, VA, USA). The culture conditions for the bEnd.3 cells followed the manufacturer’s guidelines. The cells were cultured in Dulbecco’s modified Eagle’s medium (DMEM, Gibco, Carlsbad, CA, USA) supplemented with 10% foetal bovine serum (FBS, Sigma-Aldrich, Germany) and 1% penicillin and streptomycin. The cells were treated with human recombinant IL-8 (IL-8, Thermo Fisher Scientific, Pittsburgh, PA, USA; 50 ng/ml) in 6-well plates until reaching 70% confluence. The cells were then harvested after 3 h for RT-PCR and Western blotting.

As the IL-8-mediated proliferation of endothelial cells has been reported to be dose and time dependent^13^, variable harvesting times and titres were tested in cells cultured in DMEM with FBS. The results showed the greatest cell viability under an IL-8 dose of 50 ng/ml (Supplementary Fig. S7).

To determine the role of the IL-8 receptor CXCR2 on the IL-8-downstream angiogenic pathway, bEnd.3 cells were transfected with small interfering RNAs (siRNAs) specific to CXCR2 manufactured by Thermo Fisher (PA, USA). CXCR2 siRNA sequences targeting the sense strand, 5′-GAACCAAGCUGAUCAAGGATT-3′, and the antisense strand, 5′-UCCUUGAUCAGCUUGGUUCTC-3′, were used. Appropriate concentrations (approximately 60–80%) of bEnd.3 cells cultured in DMEM for 24 h were plated in 6-well plates. Then, siRNA targeting CXCR2 was transfected at a 90 pmol concentration using Lipofectamine 2000 (Invitrogen, Carlsbad, CA, USA) according to the manufacturer’s instructions. After 24 h of incubation, IL-8 was added. The knockdown of CXCR2 in bEnd.3 cells was determined via RT-PCR.

To determine whether intracellular signalling through the p-p38 pathway is critical for IL-8-mediated angiogenesis *in vitro*, mouse endothelial bEnd.3 cells were treated with the p38 inhibitor SB203580 (50 μM) 30 min prior to IL-8 (50 ng/ml) administration. Cells were harvested at 12 h post-IL-8 treatment, as maximal angiogenic gene expression was observed at 12 h post-IL-8 administration.

### Immunocytochemistry

The facilitation of intranuclear NFκB translocation, as a component of angiogenic signalling was demonstrated in bEnd.3 cells exposed to 24 h of OGD prior to IL-8 (50 ng/ml) treatment. For hypoxic cell conditioning, cells were subjected to 24 h of OGD under 5% CO_2_ and 95% N_2_ prior to human recombinant IL-8 (50 ng/ml) exposure. The cellular response after OGD was observed by comparing the results of treatment with phosphate-buffered saline (PBS) as a control to those of IL-8 treatment; the two cell groups were harvested at the identical time point of 12 h posttreatment. Cells plated in 2-well chamber slides were rinsed twice with PBS and then fixed in 4% paraformaldehyde. After additional rinses in PBS, the cells were blocked with PBS in 2% normal goat serum with 1% Triton X-100 blocking solution for 1 h. Primary antibodies specific to p65 were used as a marker of NFκB expression. A mounting solution with 4′, 6-diamidino-2-phenylindole (DAPI) was employed. A fluorescence microscope (Eclipse Ts2, Nikon Inc., Tokyo, Japan) was used to observe and photograph the cells.

### Immunohistochemistry

Mice treated with either IL-8 or hUCBCs 7 days after HI were dissected 14 days after injury. Isoflurane anaesthetized mice were intracardially perfused with 30 ml of saline followed by 30 ml of 4% paraformaldehyde. Their dissected brains were fixed overnight and cryoprotected in 30% sucrose. Coronal sections (10 µm) were serially collected throughout the brain and mounted on glass slides: every slide was assessed for histology and IHC.

The tissues were then washed in PBS and incubated for 1 h in blocking solution (2% normal goat serum, 0.5% Triton, PBS). The tissues were subsequently incubated for 24 h with the following primary antibodies: rabbit anti-CD31 (1:50, Abcam) and mouse anti-VEGF (1:100, Novus Biologicals). Thereafter, fluorescence-conjugated secondary antibodies (1:1,000, Alexa 488-conjugated goat anti-rabbit, Alexa Fluor 594 goat anti-mouse) were incubated with the tissue sections for 1 h at room temperature. After the sections had been washed, they were mounted in ProLong Gold reagent with DAPI (Molecular Probes, Invitrogen). To estimate vessel density, the number of vessels formed was measured using the ImageJ program after an image was obtained by fluorescence microscopy at 200× magnification.

### Behavioural study

The neurological function of mice was assessed using the mNSS and the cylinder test^54^. Neurological function was graded on a scale of 0–14 (normal score: 0; maximal deficit score: 14). The cylinder test was performed to assess the functional asymmetry of the forepaw. Mice were placed in a transparent Plexiglas cylinder (diameter: 20 cm, height: 30 cm), and the number of contacts of each forepaw with the cylinder wall was counted until a total of 20 contacts were observed. The asymmetry score was calculated based on the formula below.

$$Asymmetry\,score=\frac{impaired\,forepaw\,contact\,no.+\left(\frac{bilateral\,contact\,no.}{2}\right)}{{\rm{total}}\,({\rm{impaired}}+{\rm{non}}-{\rm{impaired}}+{\rm{bilateral}})\,contact\,no.}\times 100( \% )$$

### Cresyl violet staining

Mice sacrificed after behavioural tests at 5 weeks after HI were dissected in the same manner indicated above. Coronal brain sections of 10 µm were prepared using a cryostat and stained with 0.5% cresyl violet. Images of each cresyl violet-stained brain slice was obtained under a light microscope (Nikon, Japan). The number of cells was counted for determination of cell viability using the ImageJ program. For infarct volume, the infarct area in the affected hemisphere was measured in comparison to that of contralateral hemisphere in the identical location by ImageJ program.

### Statistical analysis

The significance of the data was determined via one-way ANOVA and Tukey’s post hoc test for multiple comparison analysis using SPSS software (IBM, Chicago, IL, USA) and Prism 8.0 (GraphPad Software Inc., San Diego, CA, USA). A value of *p* < 0.05 was considered significant.

## Supplementary information

Supplementary Information.
