# Supplementary material for: Therapeutic mechanism of cord blood mononuclear cells via the IL-8-mediated angiogenic pathway in neonatal hypoxic-ischaemic brain injury
Source: Sci Rep. 2020 Mar 10;10:4446. doi: 10.1038/s41598-020-61441-0 (PMC7064601; doi:10.1038/s41598-020-61441-0)
Supplement: Supplementary file 1 — Supplementary Information. [file 41598_2020_61441_MOESM1_ESM.pdf]

Supplementary information

Therapeutic mechanism of cord blood mononuclear cells via the IL-8-mediated angiogenic pathway in neonatal hypoxic-ischaemic brain injury

Kye Hee Cho[1], Jee In Choi[2], Jin-Ock Kim[3], Joo Eun Jung[4], Dong-Wook Kim[5], MinYoung Kim[2, 6]

[1]Department of Rehabilitation Medicine, CHA Gumi Medical Center, CHA University, Gumi, Gyeongsangbukdo, Republic of Korea. [2]Rehabilitation and Regeneration Research Center, CHA University, Seongnam, Republic of Korea. [3]College of Pharmacy, Ajou University, Suwon, Gyeonggi-do, Republic of Korea. [4]Department of Neurology, University of Texas Health Science Center at Houston, McGovern Medical School, Houston, Texas, USA. [5]Department of Physiology, Yonsei University College of Medicine, Seoul, Republic of Korea. [6]Department of Rehabilitation Medicine, CHA Bundang Medical Center, CHA University, Seongnam, Gyeonggi-do, Republic of Korea.

Correspondence and requests for materials should be addressed to MYK (email: kmin@cha.ac.kr).

Supplementary Methods and Results

As potential downstream signals of the IL-8-mediumted angiogenic pathway, mitogen activated protein kinases known to be involved in angiogenic processes, including the serine/threonine-specific protein kinase Akt and extracellular signal regulated kinase (Erk)1/2 were also evaluated. However, phosphorylated (p-) Akt and p-Erk 1/2 did not show significant changes after cord blood administration in the mouse brain following hypoxic ischaemic brain injury (HI) (Supplementary Fig. S1).

The intranuclear translocation of p65, the NFκB subunit, was promoted by IL-8 treatment in mouse brain endothelial bEnd.3 cells under oxygen glucose deprivation. This finding suggests that NFκB activation was elicited by the IL-8-mediated pathway (Supplementary Fig. S2).

To find out whether the angiogenic effect of hUCBC occurs confined to the affected brain hemisphere, the unaffected contralateral hemispheres of mice were also sectioned for immunohistochemistry analysis. The upregulation of VEGF and CD31 was not observed in the contralateral hemisphere. Moreover, vessel density observed in the sham group was similar to all other groups of HI, hUCBC and IL-8 treatment, indicating that the sound side hemisphere was not affected by HI or systemic infusion of hUCBC (Supplementary Fig. S3).

To determine whether IL-8 has an effect on angiogenic processes *in vitro*, zymography was performed in bEnd.3 cell cultures. Endothelial cells were cultured in DMEM without FBS. After 24 h, the culture supernatant was harvested, and the conditioned medium was diluted so that the samples exhibited the same protein concentration. Samples (10 μL protein) in nonreducing tris-glycine sample buffer with 4% SDS were subjected to electrophoresis in a

7.5% acrylamide gel containing gelatine. The gels were washed repeatedly with washing buffer and then rinsed for 10 min in incubation buffer. The enzymatic activity of MMP-9 was significantly increased under treatment with 50 ng/mL of IL-8 (Supplementary Fig. S4).

To confirm the angiogenic effect of IL-8, actual tube formation was examined *in vitro* using mouse brain endothelial bEnd.3 cells prior to IL-8 treatment. The wells of 24-well culture cluster dishes were coated with Corning Matrigel Matrix solution (0.289 ml/well) (Corning Incorporated, MA), which was then allowed to solidify for 1 h at 37 °C. Cells (1.2×10^5 cells/well) were subsequently seeded onto Matrigel with or without 50 ng/ml IL-8. The plates were incubated for 8 h, and the number of tubes formed was then counted from 9 microscopic fields selected and photographed at random. The number of tubes formed was significantly greater in cells treated with IL-8 (50 ng/ml) (Supplementary Fig. S5).

To determine the initial time point of CXCR2 upregulation, real-time PCR was performed on brain samples from HI mice sacrificed 30 min after human umbilical cord blood administration. The primers for CXCR2 were CXCR2 forward, 5′-GAAATTTCGCCATGGACTTCTC-3′, and CXCR2 reverse, 5′-ACGAGCTAACAAAAGAAGGCCTT-3′. The expression of CXCR2 was consistently increased in the HI mouse brains treated with hUCBC compared to the HI-only mouse brains at identical time points (Supplementary Fig. S6A). This *in vivo* finding paralleled the observed *in vitro* CXCR2 expression after IL-8 treatment according to Western blotting. Mouse brain endothelial bEnd.3 cells were exposed to 24 h of oxygen glucose deprivation prior to IL-8 treatment. CXCR2 expression was continuously increased in cells treated with IL-8 compared with that in cells without IL-8 (Supplementary Fig. S6B).

To determine the dosage of IL-8 to be used in mouse endothelial bEnd.3 cells, cell viability after IL-8 administration was tested. Cells cultured in Dulbecco’s modified Eagle’s

medium with 10% foetal bovine serum were treated with various dilutions of IL-8 (1, 10, 50, and 100 ng/ml). Cell viability at 24 h post-IL-8 treatment was increased significantly under treatment with up to 50 ng/ml of IL-8 (Supplementary Fig. S7).

Individual full-length blots are included in the Supplementary Data in the end.

Supplementary Figures with Legends

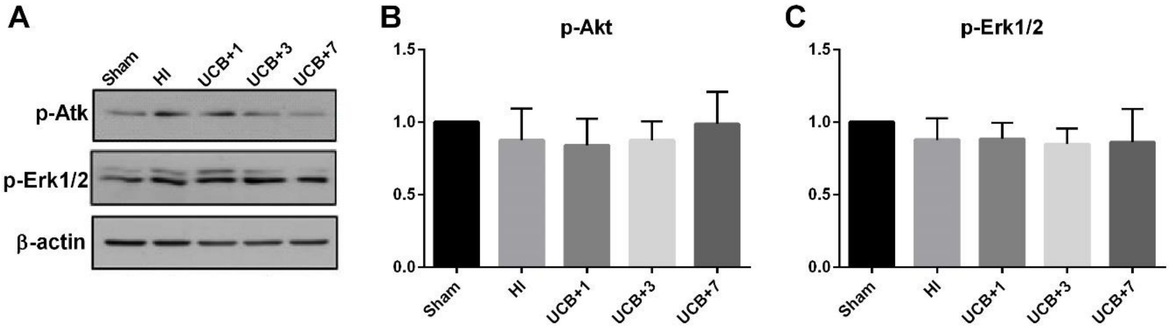

Supplementary Fig. S1. (A) Western bolts of p-Akt and p-Erk1/2 in the HI mouse brain following the administration of human cord blood. (B-C) The graphs depict the band intensity in each Western blot. The data were collected from triplicate results. Error bars indicate standard error (n = 4).

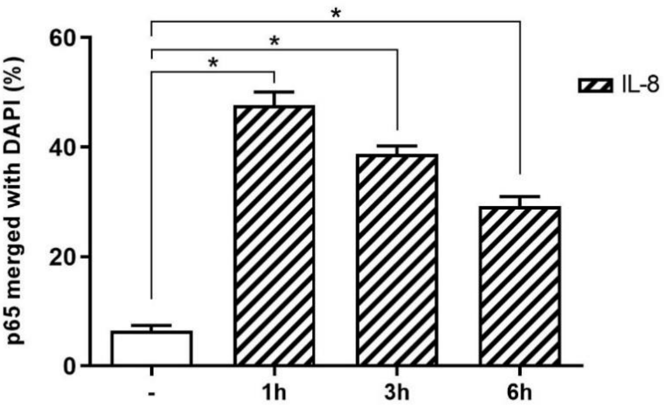

Supplementary Fig. S2. The immunocytochemistry of mouse endothelial bEnd.3 cells revealed that the proportion of p65 intranuclear translocation was significantly increased in a time-course analysis after IL-8 administration as shown in the graph. Error bars indicate the standard error (n = 4).

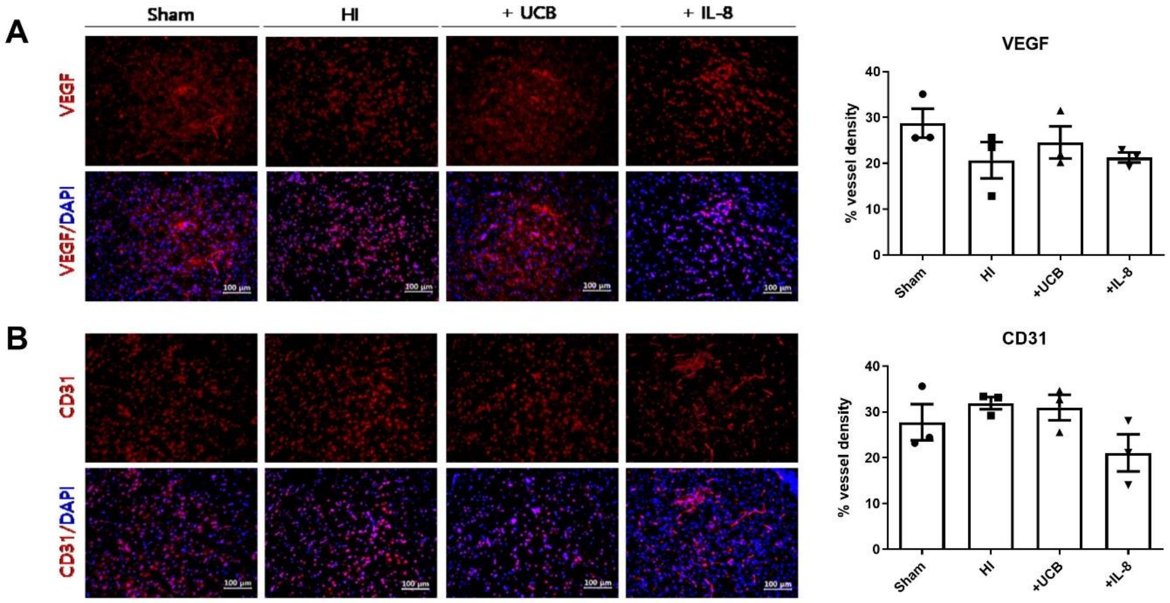

Supplementary Fig. S3. In the striatum of unaffected hemisphere, contralateral to the affected hemisphere, immunohistochemistry staining was performed for VEGF and CD31. (A) The graph depicts the density of vessels (percentage) expressing VEGF among cells stained with DAPI. (B) The graph depicts the density of vessels (percentage) expressing CD31 among DAPI-stained cells. No statistical differences were observed between groups. Data are shown as the mean ± SEM (n = 3). HI, hypoxic-ischaemic brain injury; DAPI, 4′, 6-diamidino-2-phenylindole for nuclear staining; +UCB, HI mice subjected to hUCBC treatment; +IL-8, HI mice subjected to IL-8 treatment; hUCBCs, human umbilical cord blood mononuclear cells

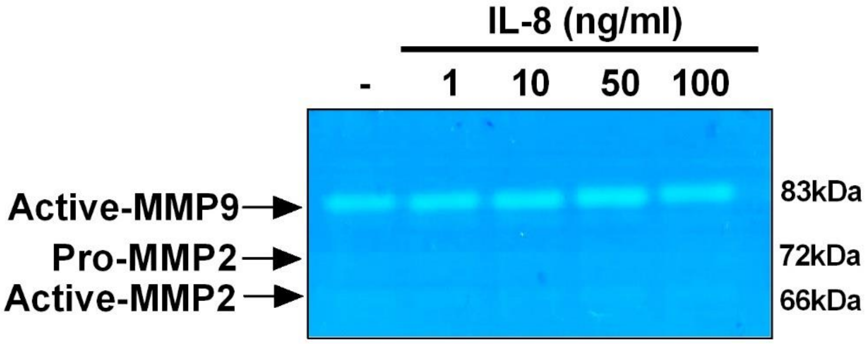

Supplementary Fig. S4. MMP-9 activity after IL-8 treatment showed the greatest increase at a dose of 50 ng/ml IL-8 upon zymography.

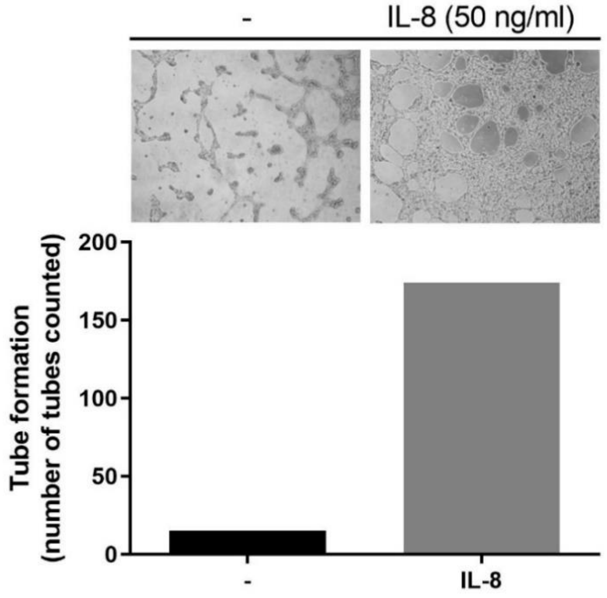

Supplementary Fig. S5. The angiogenic effect of IL-8 was confirmed in endothelial bEnd.3 cells. The number of tubes formed was much greater in IL-8 (50 ng/ml) treated cells.

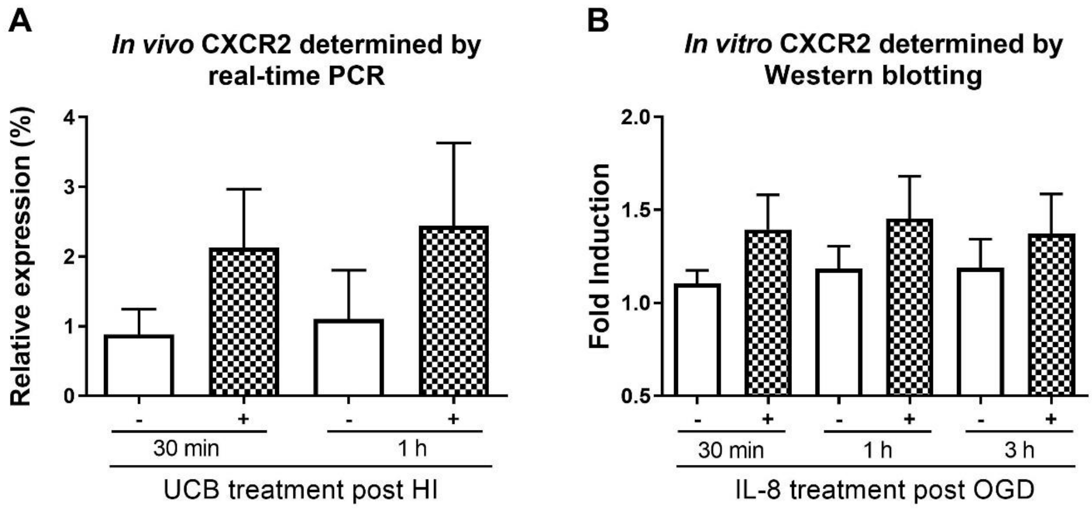

Supplementary Fig. S6. (A) CXCR2 expression was consistently increased to a greater extent in hUCBC-treated HI mouse brains than in those without hUCBC treatment according to real-time PCR. Error bars indicate standard error (*n* = 4). (B) The results of *in vivo* CXCR2 upregulation after hUCBC administration were confirmed *in vitro* in mouse brain endothelial bEnd.3 cells exposed to 24 h OGD prior to IL-8 treatment. Cells treated with IL-8 consistently expressed higher levels of CXCR2 than those not subjected to IL-8 exposure. Error bars indicate the standard error (*n* = 3). UCB, hUCBC treatment; OGD, oxygen glucose deprivation; hUCBCs, human umbilical cord blood mononuclear cells

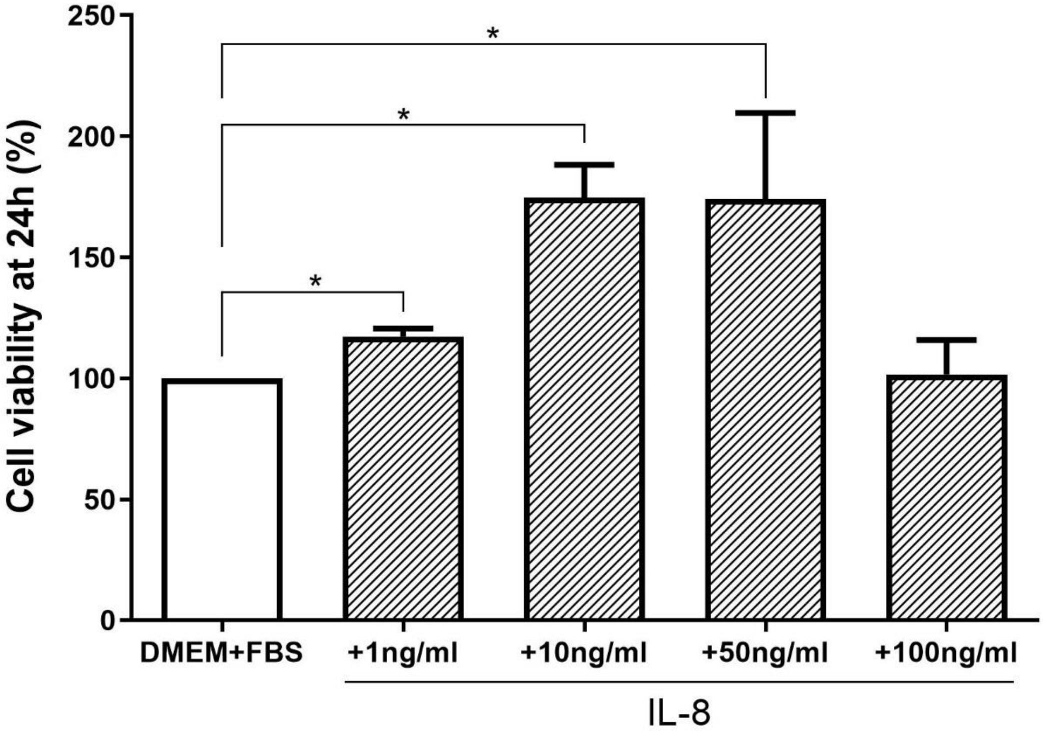

Supplementary Fig. S7. Cell viability was tested for each different dose of IL-8 applied to microvascular endothelial bEnd.3 cells. Cells treated with IL-8 were significantly more viable under treatment with up to 50 ng/ml of IL-8. Error bars indicate standard error (*n* = 3). Asterisks (*) indicate significant differences (*P<0.05, Mann-Whitney test). DMEM, Dulbecco's modified Eagle's medium; FBS, foetal bovine serum

Supplementary Data

Fig. 2 c

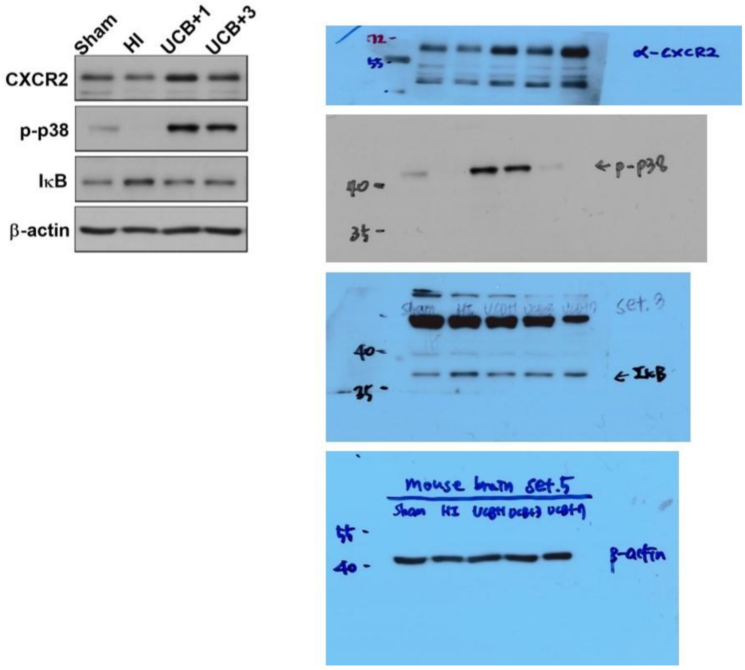

Fig. 3a

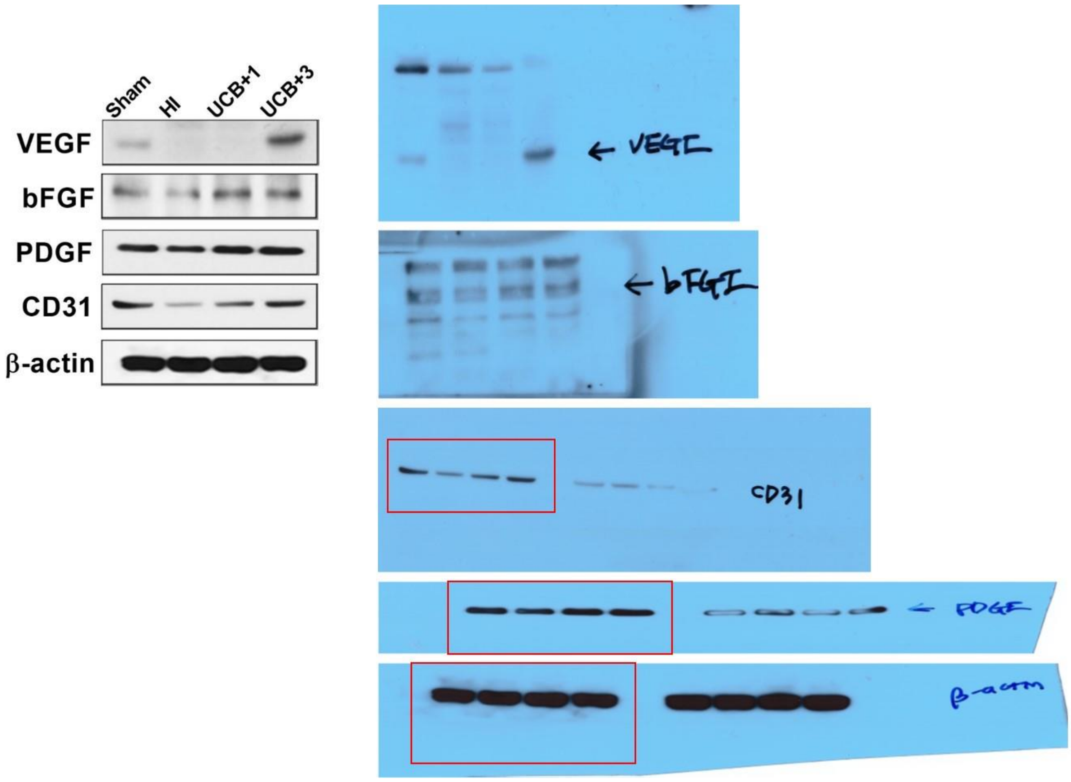

Fig. 4 a

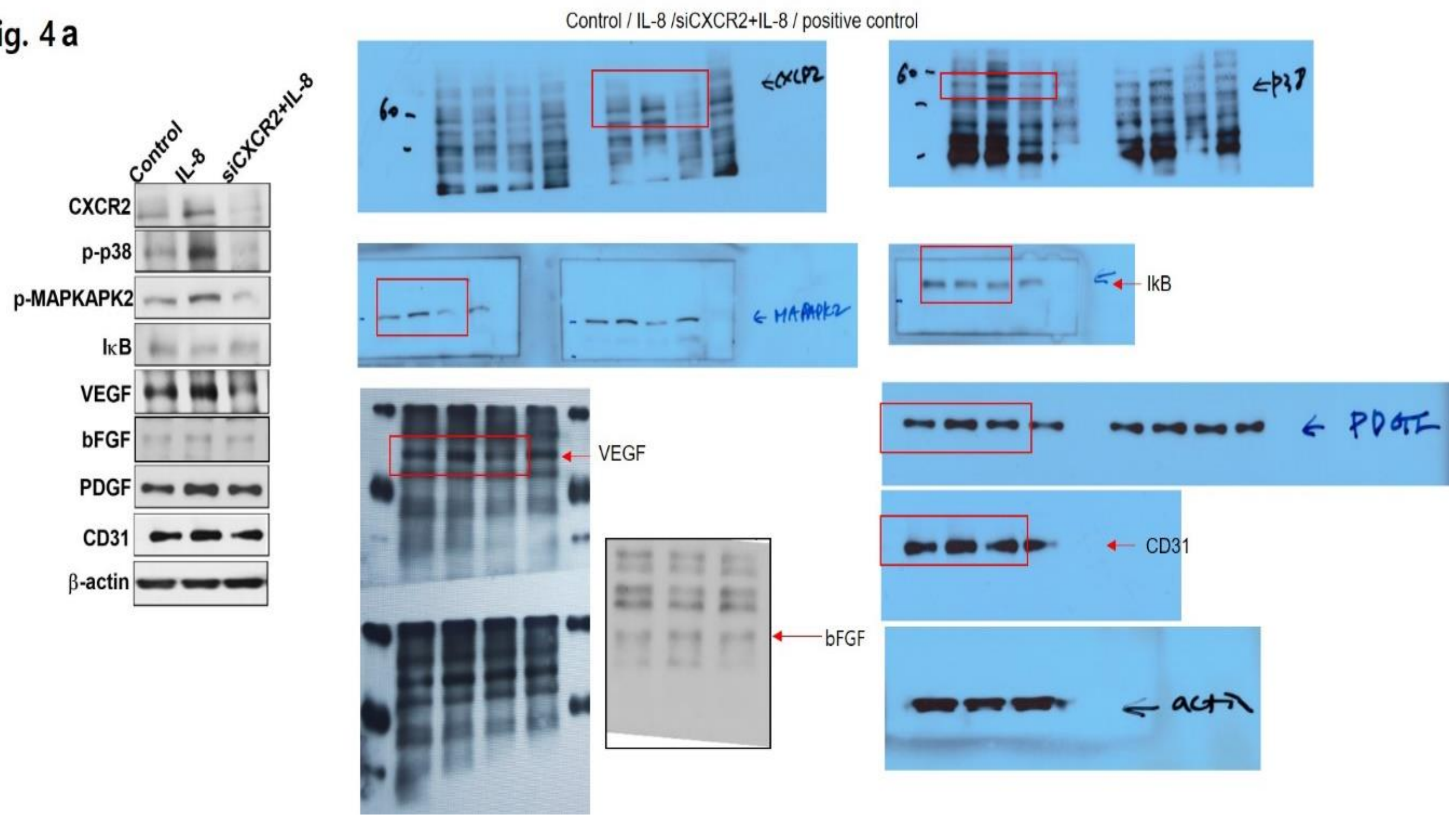

Control
IL-8
siCXCR2+IL-8
CXCR2
p-p38
p-MAPKAPK2
IκB
VEGF
bFGF
PDGF
CD31
β-actin
Control / IL-8 /siCXCR2+IL-8 / positive control
IkB
VEGF
bFGF
CD31

Fig. 5 a

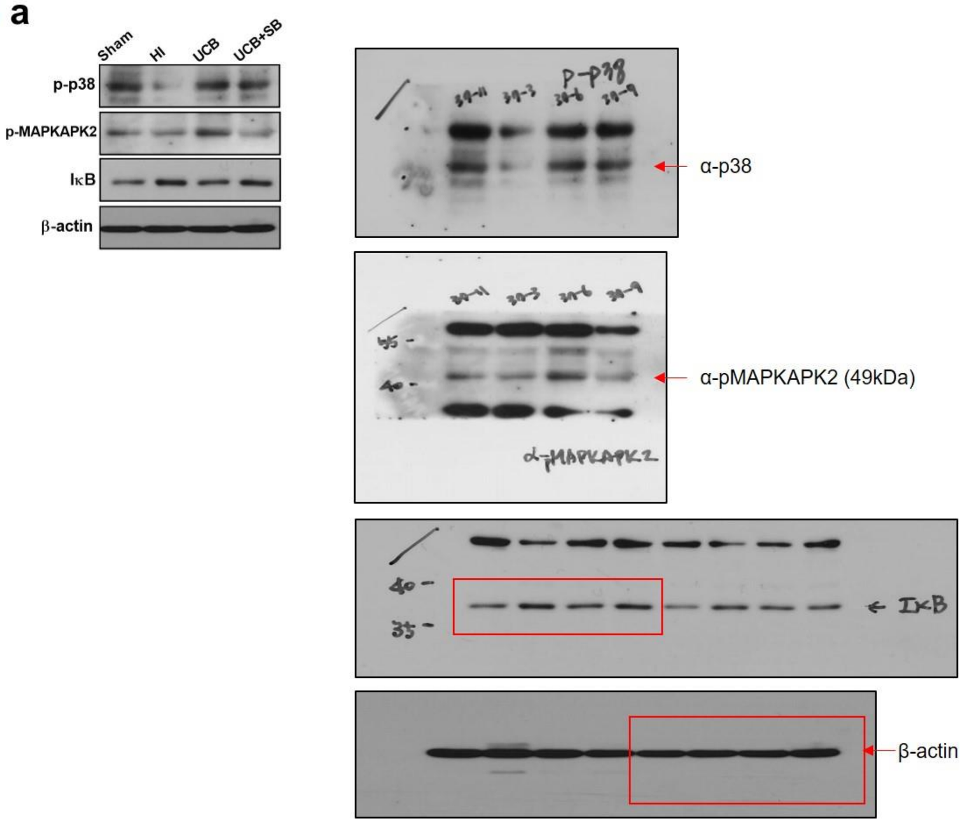

Sham
HI
UCB
UCB+SB
p-p38
p-MAPKAPK2
IκB
β-actin
p-p38
α-p38
40
35
α-pMAPKAPK2 (49kDa)
40
35
IκB
β-actin

Fig. 5 c

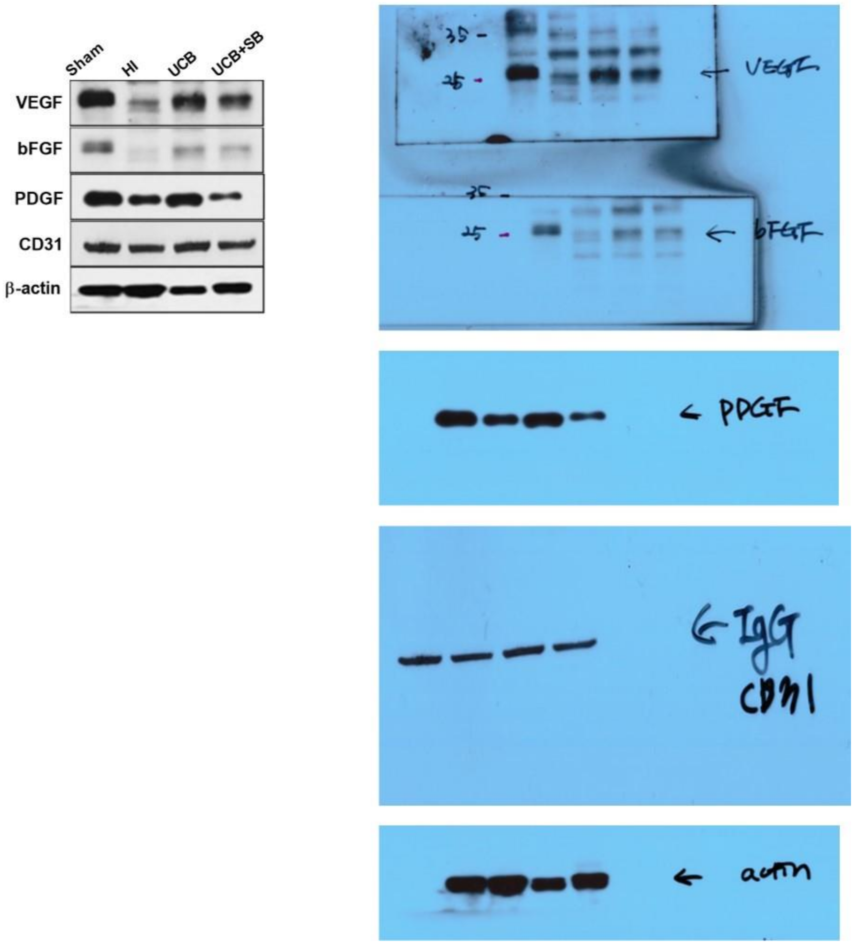

Sham
HI
UCB
UCB+SB
VEGF
bFGF
PDGF
CD31
β-actin
35
25
VEGF
35
25
bFGF
PDGF
IgG
CD31
actin

Fig. 6 a

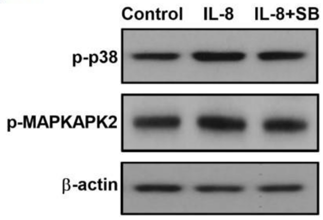
Control
IL-8
IL-8+SB
p-p38
p-MAPKAPK2
β-actin

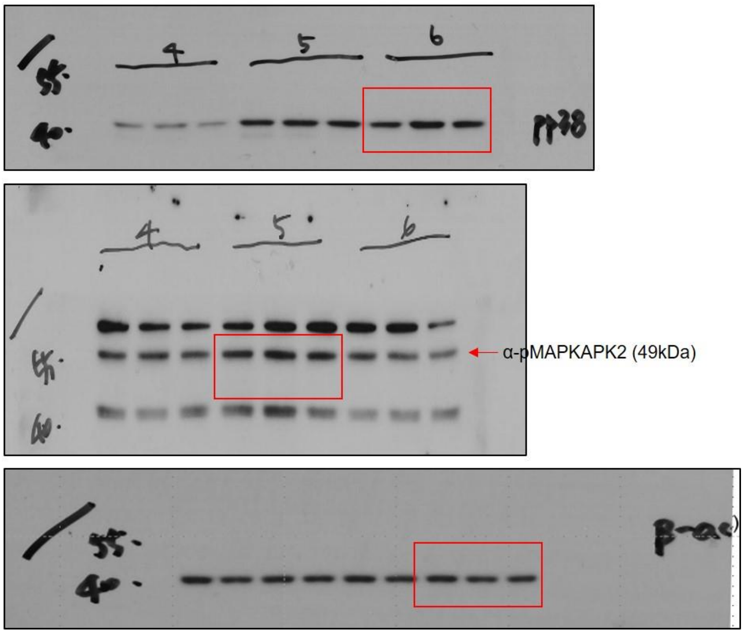
4
5
6
55
40
p38
α-pMAPKAPK2 (49kDa)
β-actin

Fig. 6 e

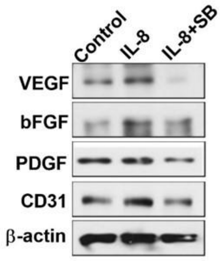
Control
IL-8
IL-8+SB
VEGF
bFGF
PDGF
CD31
β-actin

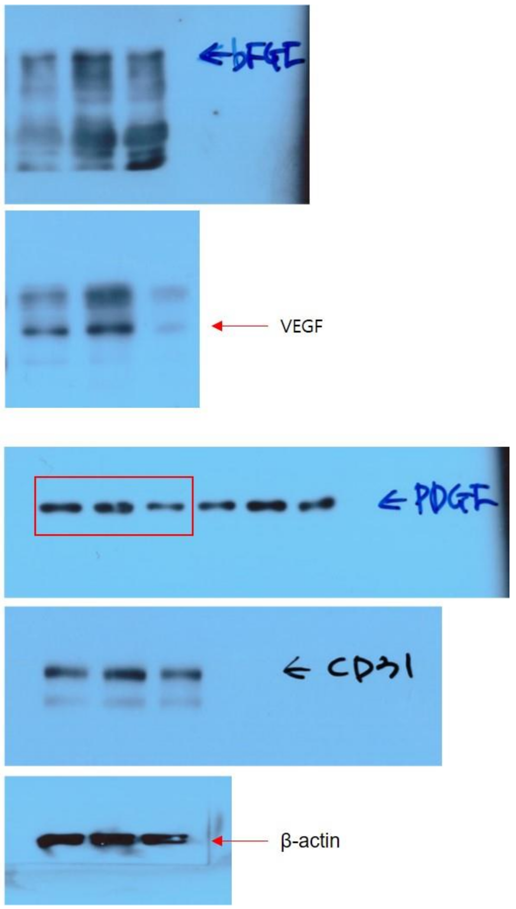
← bFGF
VEGF
← PDGF
← CD31
β-actin
